# The Hidden Threat of Microplastics in Traditional Cigarettes: A Narrative Review of Health and Environmental Risks

**DOI:** 10.3390/jcm14113721

**Published:** 2025-05-26

**Authors:** Justyna Śniadach, Aleksandra Kicman, Sylwia Szymkowiak, Napoleon Waszkiewicz

**Affiliations:** 1 Department of Psychiatry, The Faculty of Medicine, Medical University of Białystok, 15-272 Białystok, Poland; sniadach.justyna@gmail.com (J.Ś.); olakicman@gmail.com (A.K.); 2 HCP Medical Center in Poznań, 61-485 Poznań, Poland; szymkowiaksylwia@gmail.com

**Keywords:** microplastic, pro-inflammatory cytokines, oxidative stress, classic cigarettes, cigarette filters, plastic pollution

## Abstract

Exposure to microplastics (MPs) in biological systems can lead to particle toxicity, oxidative stress, and inflammatory changes, potentially contributing to cancer development, digestive disorders, respiratory issues, and fertility problems. Traditional cigarette users are particularly vulnerable, as MPs have been detected in 99% of cigarette filters, exposing smokers to these particles through inhalation and ingestion. This narrative review aims to analyze the sources, health implications, and biochemical impact of MPs derived from cigarette consumption. A literature search was conducted using databases such as PubMed, Scopus, and Google Scholar, applying strict inclusion criteria: peer-reviewed studies published between 2010 and 2025 and keywords such as “microplastics”, “smoking”, “tobacco”, “oxidative stress”, “pro-inflammatory cytokines”, “cell viability”, “regulatory policies”, and “health effects”. Findings indicate that MPs are present in biological samples such as blood (77% of cases), placenta (75%), lung tissue (88%), and feces (100%), demonstrating systemic human exposure. The environmental implications of improper cigarette disposal further exacerbate the global microplastic crisis. This review highlights potential mitigation strategies to reduce the health and environmental impact of cigarette-derived microplastics.

## 1. Introduction

Microplastics are plastic particles, films, and fibers with a diameter of 1–5 µm. Cigarette filters, often composed of cellulose acetate, are a significant source of microplastic pollution in the environment. When discarded improperly, these filters degrade into smaller plastic particles that persist in ecosystems for years [1,2,3]. These pollutants pose a serious threat to aquatic and terrestrial life, entering food chains and causing harm through physical and chemical pathways [1,2,3,4].

Globally, cigarette consumption contributes significantly to environmental pollution. Each year, approximately 4.5 trillion cigarette butts are discarded, making them the most frequently littered item worldwide. These filters, composed of non-biodegradable plastics, can take up to 10 years to decompose, releasing toxic chemicals into soil and waterways [5]. Additionally, tobacco farming leads to deforestation of nearly 200,000 hectares annually, while cigarette production consumes 22 billion tons of water and emits 84 million tons of CO_2_, exacerbating climate change and resource depletion [5].

Despite growing concerns about microplastic contamination, research on their direct impact on human health—particularly from cigarette-derived microplastics—remains limited. Studies suggest that cigarette smoke may contain microplastics originating from filter degradation, potentially affecting respiratory health [6,7]. The scientific community continues to investigate the extent of this contamination and its implications for human well-being and environmental sustainability [8,9].

This review aims to systematically analyze the health risks associated with cigarette-related microplastics, focusing on their role in inflammation, oxidative stress, and toxicity [10,11,12]. Unlike previous studies, which primarily examine cigarette waste as an environmental pollutant, this work explores the biochemical interactions between cigarette-derived microplastics and human tissues, emphasizing potential consequences for smokers.

Understanding how cigarette-derived microplastics contribute to health issues is essential in addressing their broader impact. Comprehensively assessing the sources, exposure pathways, and associated health risks is crucial, bridging the gap between environmental contamination research and human health studies.

## 2. Materials and Methods

This narrative review was conducted through a comprehensive literature search using PubMed, Scopus, and Google Scholar as well as other available sources. The following keywords were applied: “microplastics”, “smoking”, “cigarettes”, “tobacco”, “oxidative stress”, “pro-inflammatory cytokines”, “cells viability”, policy”, and “health effects”.

This article synthesizes the existing literature on the relationship between microplastics, smoking, and inflammation. A comprehensive search of databases was conducted using keywords. Studies published in peer-reviewed journals from 2010 to 2025 were included to ensure the relevance and recency of findings.

The review focuses on studies that examine the sources of microplastics in cigarettes, their health implications, particularly concerning respiratory, digestive, and inflammatory effects, and the environmental impact of microplastic pollution. Additionally, policy and regulatory measures were reviewed to identify potential strategies for mitigating the impact of microplastics from smoking products.

To ensure methodological rigor, a structured selection process was applied, incorporating predefined inclusion and exclusion criteria. A detailed description of these criteria and the approach to article selection is provided below.

### 2.1. Inclusion Criteria

To ensure the relevance and reliability of included studies, the following criteria were applied:

- Timeframe: studies published between 2010 and 2025 to reflect recent scientific findings.
- Relevance: studies addressing microplastics in cigarette filters, their health effects, or their biochemical interactions with human tissues.
- Biological impact: research focusing on oxidative stress, inflammation, and toxicity due to microplastic exposure.
- Regulatory aspects: articles discussing policy and legal measures aimed at mitigating microplastic pollution from smoking products.
- Study design: both human and animal studies investigating microplastic accumulation in tissues (e.g., lungs, blood, placenta).

### 2.2. Exclusion Criteria

Studies that did not provide sufficient data for assessing microplastic exposure pathways and associated health risks were also excluded:

- Non-peer-reviewed sources, editorials, and opinion-based papers.
- Studies focusing solely on general environmental microplastic pollution without human health implications.
- Laboratory-based in vitro studies lacking direct relevance to human exposure.
- Articles with insufficient methodological transparency or those flagged for a high risk of bias.

### 2.3. Article Selection and Bias Minimization

The initial search yielded 560 articles, which were independently screened by two reviewers, evaluating titles and abstracts for relevance to the study objectives. Following this screening, 195 full-text articles were reviewed in detail. Final inclusion was determined through a structured selection process, ensuring objectivity and methodological rigor. Any discrepancies in selection were resolved through discussion or consultation with a third reviewer.

Ultimately, 80 articles were included in this review, reflecting the most relevant and high-quality studies on microplastics in cigarette filters, their health effects, biochemical interactions, and regulatory aspects.

This process was applied to assess study quality and ensure methodological rigor. Additionally, standardized data extraction procedures were implemented to maintain consistency across selected articles and minimize variability in reported findings. Studies were carefully screened for relevance, with priority given to those incorporating clinical evidence, regulatory perspectives, and public health surveillance measures. This methodological approach ensures a balanced and interdisciplinary analysis, integrating insights from clinical, toxicological, environmental, and regulatory domains to provide a comprehensive understanding of microplastic exposure and its implications.

**Figure 1:**
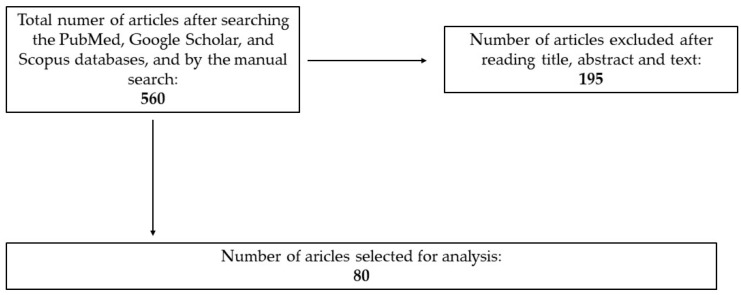
Selection process of articles for review.

The selection process is visually represented in Figure 1, which outlines the systematic approach used to filter and finalize the included studies.

## 3. Sources of Microplastic Exposure and Health Implications

Microplastics are plastic particles ≤ 5 µm. Sources of microplastics in the environment include material raw materials, paints, personal care items, washing wastewater, food, clothing, and peri-medical sources [13]. Based on the source of origin, they are divided into primary microplastics—intentionally produced for industrial use or various types of use—and secondary microplastics, which are the result of breakdown by photo-oxidation and thermo-oxidation processes of larger parts of plastic into smaller particles. Their source is improperly stored plastic waste [13,14,15].

Another division of microplastics is based on their shape; microplastics in the shape of fibers, granules, films, sponges, foams, and microspheres are distinguished [15]. Microplastics can be spread by wind and water, which is why they are found in all environments—from the surface to the bottom of the seas and oceans, in agricultural fields, in soil at every altitude, and also in ice and the waters of lakes and rivers. MP has been detected in 1300 animal species, including humans [2,3,5,13,14]. The exact impact of microplastics on human health is not clearly understood—on the one hand, it can directly exert adverse effects on human health by accumulating in organs and cells and by absorbing toxic substances that are released in the human body [2,13].

### 3.1. The Gastrointestinal Route

Ingestion is considered the main route of human exposure to microplastics [14]. Due to the high plastic pollution of the marine ecosystem, fish and seafood are the main sources of these compounds [15]. Other microplastic-containing products include salt, honey, sugar, and bottled water [14]. Food processing and packaging are also associated with microplastic contamination of food [16]. It is estimated that annually a person consumes an amount of microplastic equivalent to 50 plastic bags per year or one credit card per week [17]. Importantly, the presence of microplastic is found in gastrointestinal organs such as the colon and liver [18].

Damage to the digestive system by microplastics begins in the mouth, where these particles alter the composition of the oral microbiota. With the participation of saliva, microplastic compaction can occur [19]. Within the intestines, microplastics disrupt the normal microflora of these organs, thereby leading to intestinal inflammation and related diseases such inflammatory bowel disease. Importantly, microplastics induce oxidative stress, which is also associated with the development of inflammation and apoptosis of epithelial cells [20]. Chronic microplastic-induced inflammation is also observed in the liver; MP affects hepatocyte structure and metabolism. It has been suggested that MP is associated not only with processes leading to cirrhosis but may also be a cause of acute liver injury [19,20,21].

### 3.2. Inhalation Route

Microplastics are deposited in the airways and lungs, and the degree of exposure depends on their size—their presence is also found in sputum, bronchoalveolar lavage (BAL), and lung cancer biopsy specimens. Particles less than 10 μm in diameter have the ability to penetrate the bronchi, while fractions finer than 2.5 μm and ultrafine particles can reach the alveoli. The researchers discovered 39 microplastic particles in 11 of 13 lung tissue samples. The researchers found a total of 0.23 particles per gram of tissue in the upper lungs, 0.33 in the middle, and 1.65 in the lower lungs. Penetrating the airway epithelium, microplastic particles induce local inflammation, which leads to lung tissue damage [22,23,24,25,26]. In rats inhaling microplastic particles, pro-inflammatory and alveolar destruction is found [27]. While the precise role of microplastics in respiratory disease development remains unclear, evidence suggests that inhalation of microplastic-containing air may contribute to respiratory discomfort and inflammation. However, since it is the lungs that accumulate the largest amounts of microplastics [24], further research is needed on their role in the development of lung diseases.

### 3.3. Cutaneous Route

MP is absorbed through the pores and is found on the skin of the face, lips, and hands, as well as in hair. It has been shown to induce skin lesions, provoke inflammatory reactions, and disrupt homeostasis of physiological skin functions. Sources of MP exposure include cosmetics and other products for skin application and, to a lesser extent, contact with microplastic-contaminated water. Damaged skin allows slightly larger particles of MP to penetrate [28,29]. In smokers, the penetration of microplastic is mainly through the skin of the mouth and through smoke settling on the skin, even in passive smokers. This is also due to the irritant effect of nicotine. The most important sources and routes of microplastic absorption are shown in Figure 1.

### 3.4. Potential Carcinogenic Risks of Microplastic

Microplastics are increasingly recognized as potential carcinogens due to their ability to bioaccumulate in human tissues and interact with cellular processes that may lead to mutations and oxidative stress [30]. Studies have indicated that microplastics can act as carriers of carcinogenic chemicals, such as phthalates, bisphenols, and chlorinated paraffins, which may contribute to DNA damage and disruptions in cell cycle regulation [31]. Recent studies have provided compelling evidence linking microplastic exposure to various carcinogenic mechanisms. Microplastics have been detected in 80% of the lung tissue samples from patients undergoing surgery for lung cancer, raising concerns about their potential role in pulmonary carcinogenesis [32]. Additionally, exposure to microplastics has been associated with a 35% increase in reactive oxygen species (ROS), a critical factor in oxidative stress, which can lead to DNA damage and mutations contributing to tumor initiation [31].

Beyond oxidative damage, microplastics have also been implicated in inflammatory responses. Chronic exposure has been correlated with a 2.5-fold increase in pro-inflammatory cytokines, such as IL-6 and TNF-α, which are well-documented contributors to tumor progression through sustained inflammation and immune system modulation [30]. Moreover, microplastics possess endocrine-disrupting properties, mimicking certain chemicals that interfere with hormonal regulation, leading to a 40% increase in abnormal cell proliferation in hormone-sensitive tissues, such as the liver and breast, potentially increasing the risk of hormone-related cancers [31].

These findings highlight the urgent need for further investigation into the long-term carcinogenic effects of microplastics, as well as the development of standardized biomonitoring methods to assess human exposure and mitigate potential health risks.

### 3.5. Effects of Microplastics on Reproductive Health

MP particles affect fertility, both male and female. The presence of MP is found in breast milk, placenta, and male and female reproductive organs. MP negatively affects the hypothalamic–pituitary–gonadal axis, thereby causing endocrine disruption and resulting fertility disorders. In women, MP causes placental dysfunction, endometrial hypertrophy and fibrosis, and atrophy of ovarian tissue. For men, the most important negative effects of MP are related to sperm damage through MP’s induction of oxidative stress. MP also affects decreased fertility by damaging the blood–nucleus barrier. MP has also been shown to decrease the viability of both sperm and oocytes. Moreover, MP may be involved in the formation of embryonic and fetal disorders, thereby contributing to premature birth or fetal malformations [33,34,35,36,37,38,39].

## 4. Cigarette Filters as Microplastic Sources

**Figure 2:**
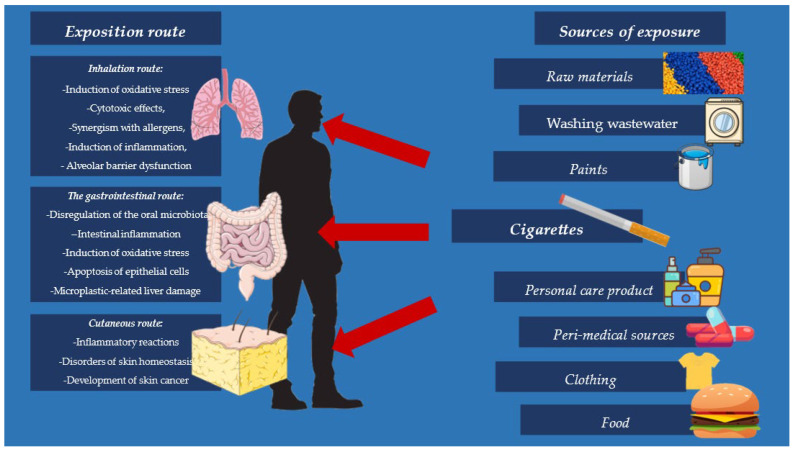
Routes and sources of microplastic exposure in the human body.

**Figure 3:**
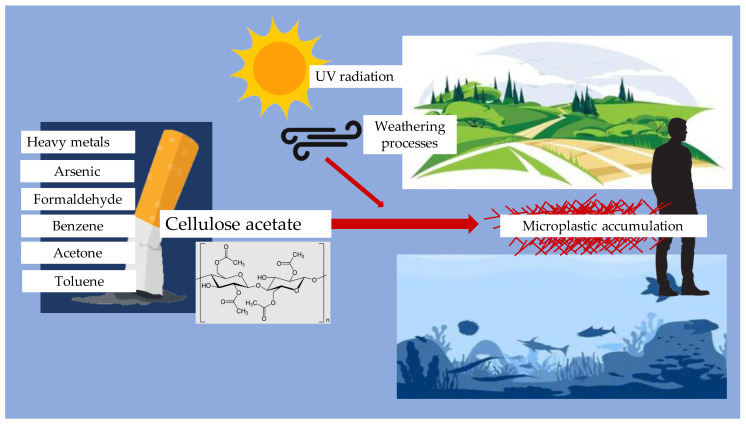
Formation and accumulation of microplastic from cigarette filters.

Cigarette filters are primarily made of cellulose acetate, a type of plastic that is not biodegradable. The improper disposal of cigarette butts contributes significantly to microplastic pollution. Studies have shown that cigarette butts can leach harmful chemicals into the environment, including heavy metals and toxic substances, which can adversely affect aquatic life and ecosystems [40,41]. Burnt filters can release about 0.3 million tons of microfibers per year. The leachate from cigarette butts has been found to be toxic to various marine organisms, including fish and invertebrates, raising concerns about the broader ecological impacts of cigarette waste [41,42]. Moreover, the breakdown of cellulose acetate filters into microplastics can occur through environmental weathering processes, such as UV radiation and moisture exposure. Research indicates that these microplastics can persist in the environment for years, contributing to the accumulation of plastic pollution in terrestrial and aquatic ecosystems [43,44,45]. The presence of microplastics in the environment poses a risk not only to wildlife but also to human health, as these particles can enter the food chain and potentially accumulate in human tissues [40,41,42,43]. The chain of microplastic turnover from filters is shown in Figure 2 and the Formation and accumulation of microplastic from cigarette filters is shown in Figure 3.

## 5. Effect of Microplastics on Inflammatory Cytokines

The exact effect of microplastic on the production of pro-inflammatory cytokines is currently poorly studied. According to most studies, in vitro microplastic increases the production of pro-inflammatory cytokines. According to a study by Weber et al. [46], dendritic cells and monocytes under the influence of microplastic particles produce increased amounts of pro-inflammatory cytokines such as IL-6, TNF-α, and IL-10. Importantly, the release of these cytokines was dependent on the shape of the microplastic, with the highest pro-inflammatory response observed for irregular shapes compared to spherical shapes. Also, a study by Prietl et al. [47] showed an increase in IL-6 and IL-8 production by monocytes and macrophages under the influence of MPs. The work of Kwon et al. [48] showed that human microglia cells accumulate microplastic, which is associated with an increase in pro-inflammatory response (increase in TNF-α, IL-6, and IL-1β expression) and a decrease in anti-inflammatory response (decrease in TGF-β and IL-10 expression). Additionally, Prietl et al. [47] investigated how nano-sized and micro-sized polystyrene particles influence phagocyte function, contributing to the broader understanding of immune responses to microplastics. Also, epithelial cells under the influence of MPs produce higher amounts of pro-inflammatory cytokines, as demonstrated by the work of Zhang et al. [49].

Some experiments were also conducted on cells of cancer lines: A549 (human lung epithelial cell line) and HeLa (epithelial cervical cancer cell line). It has been observed that cells of the cancer line produce increased amounts of TNF-α, IL-1β, INF-y, IL-6, and IL-8 after incubation with MP molecules [50]. Similar observations were also found for AGS cells (human gastric adenocarcinoma cell line) and U937 (human pro-monocytic leukemia cell line), THP-1 (human leukemia monocytic cell line), A549, and HaCaT cells (spontaneously immortalized human keratinocyte cell line) [51,52]. A single study by da Silva Brito et al. [50] on the kidney of a human embryo also confirmed the increased production of TNF-α, IL-1β, INF-y, IL-6, and IL-8 under the influence of MPs.

The production of pro-inflammatory cytokines is also increased in the liver. The oral intake of microplastics leads to an increase in liver weight, the overall liver index, and the expression of serum and liver function-related indicators. Microplastics also heighten the infiltration of natural killer cells and macrophages into non-parenchymal liver cells while decreasing the infiltration of B cells into the same tissues. However, the infiltration of T cells into non-parenchymal liver cells is unaffected by microplastics. The ingestion of microplastics also results in an up-regulation of IFN-γ, TNF-α, IL-1β, IL-6, and IL-33 mRNA expression, but a down-regulation of IL-4, IL-5, IL-10, IL-18, and TGF-β1. These processes are regulated via the NF-κB pathway in hepatic non-parenchymal cells. In conclusion, microplastics disrupt the inflammatory process in liver tissues through the NF-κB signaling pathway [53].

Current research indicates that microplastics stimulate the production of pro-inflammatory cytokines, leading to inflammatory responses in multiple biological systems, including immune cells, epithelial tissues, cancer cell lines, and liver tissues. While most studies agree that microplastic exposure upregulates cytokine expression, differences in particle shape appear to influence the strength of the inflammatory reaction, with irregularly shaped microplastics showing higher cytokine activation than spherical ones [46].

However, contradictions exist regarding the immune regulatory balance. Some studies have reported anti-inflammatory cytokine suppression (e.g., decreased TGF-β and IL-10 levels in microglia and liver tissues), suggesting that microplastics may disrupt homeostasis, enhancing inflammation while inhibiting resolution mechanisms [47,53]. This imbalance could contribute to chronic inflammatory diseases, but further research is required to confirm these long-term effects.

These findings highlight the need for standardized methodologies to assess cytokine responses to microplastic exposure across different biological models. Additionally, investigating the threshold levels at which microplastic-induced inflammation becomes pathological will be crucial for understanding its clinical relevance [48,49,52].

## 6. Effect of Microplastics on Oxidative Stress Parameters

Microplastic particles have a significant effect on oxidation–reduction balance. In vitro studies have shown a significant increase in reactive oxygen species (ROS) production in U937 cells, THP-1, A549, HaCaT [51], Caco-2 (human colorectal adenocarcinoma cell line) [54,55], and HeLa cells [56]. In particular, large amounts of H_2_O_2_ have been found in Caco-2 cells [57]. In contrast, an increase in nitric oxide production has been observed in human monocytes and macrophages [46]. A study by da Silva Brito et al. [50] showed an increase in thiol groups under the influence of microplastics—thiol groups have antioxidant properties. It is possible that this is a protective mechanism against the action of microplastics.

Observations also relate to changes in the activity of major antioxidant enzymes under the influence of microplastic. Studies on human monocytes and macrophages showed an increase in myeloperoxidase (MPO) production [46]; a study by Salimi et al. [58] showed that human lymphocytes had elevated glutathione disulfide (GSSG) activity with a concomitant decrease in glutathione content after exposure to microplastic. In HEK293 cells (human embryonic kidney cell line), decreased activity of heme oxygenase 1 (HMOX1) was found [59]. Some of the studies were also based on the effect of microplastic on organoids of hepatic and cardiac origin. In human liver organoids, there is a significant decrease in glutathione reductase (GST) and superoxide dismutase (SOD) activities and a decrease in glutathione [60]. Human-originated cardiac organoids also show a decrease in SOD activity [61].

Research consistently demonstrates that microplastic exposure disrupts oxidative balance, leading to increased production of ROS, H_2_O_2_, and nitric oxide, all of which contribute to cellular stress and potential toxicity [51,56,57,58]. Notably, the thiol group increase observed in human embryonic kidney cells suggests a possible compensatory antioxidant response, indicating that some cells may attempt to counteract microplastic-induced oxidative damage [50].

However, significant contradictions arise in antioxidant enzyme activity. While microplastics upregulate oxidative stress markers, they also reduce key antioxidant defenses, such as glutathione content, glutathione reductase, and heme oxygenase activity [54,55,56,57,58,59,60]. This paradox suggests that while oxidative stress is heightened, the body’s ability to neutralize reactive species is simultaneously compromised, potentially exacerbating tissue damage over time.

Further investigation is required to determine threshold levels for toxicity, the long-term physiological consequences, and whether different microplastic compositions (size, shape, and material) influence oxidative stress severity differently [58,60]. Standardized methodologies will be essential for clarifying these mechanisms and assessing the clinical relevance of microplastic-induced oxidative damage.

## 7. Effect of Microplastics on Cells Viability

The cytotoxic effect of microplastic is a matter of debate. This cytotoxicity is observed at higher doses, and this effect is dependent on the type of microplastic, its concentration, and particle size. Most studies have used polystyrene particles at various concentrations (from 10 ng/mL to 100 µg/mL), where cytotoxicity of this compound has been demonstrated, especially through induction of apoptosis and necrosis in cells of cancer lines [48,59] and human-originated cardiac organoids [61]. A single study showed only weak cytotoxicity of microplastic in the form of polystyrene, a phenomenon that was observed with high concentrations of this compound (1–100 µg/mL) [55,57]. Importantly, according to some research teams, microplastic in the form of polystyrene does not show cytotoxic properties, regardless of the dose, incubation time with cells, and particle shape [56,62]. Other types of microplastic particles, such as polymethylmethacrylate (PMMA), have been shown to have a slight cytotoxic effect on cells of cancer lines and cells of kidney of a human embryo [50]. Polyethylene (PE) microparticles showed no cytotoxicity on HeLa and T98G cells (glioblastoma cell line), while polyvinyl chloride at concentrations of 24, 48, and 96 μg/mL showed cytotoxic properties on human lymphocytes [63,64]. Given such conflicting reports, it is advisable to conduct further studies determining the cytotoxicity of different forms of microplastic.

The cytotoxic effects of microplastics remain inconsistent across studies, with findings varying based on particle type, concentration, and size [49,54,56,59,60]. While polystyrene microplastics have been associated with apoptosis and necrosis in cancer cell lines and cardiac organoids, other studies suggest weak or negligible cytotoxicity, even at high concentrations [56,62].

Contradictions also arise in the toxicity of different microplastic materials. For instance, polymethylmethacrylate (PMMA) has shown moderate cytotoxic effects, whereas polyethylene microparticles demonstrated no toxicity on HeLa and T98G cells [50]. Meanwhile, polyvinyl chloride (PVC) exhibits a strong cytotoxic effect on human lymphocytes, indicating that certain plastic compounds may pose greater risks than others [64].

These variations underscore the need for standardized methodologies to evaluate microplastic cytotoxicity. Future studies should examine long-term exposure effects, interactions with biological membranes, and potential tissue-specific toxic mechanisms to fully assess the health risks associated with microplastic accumulation.

**Figure 4:**
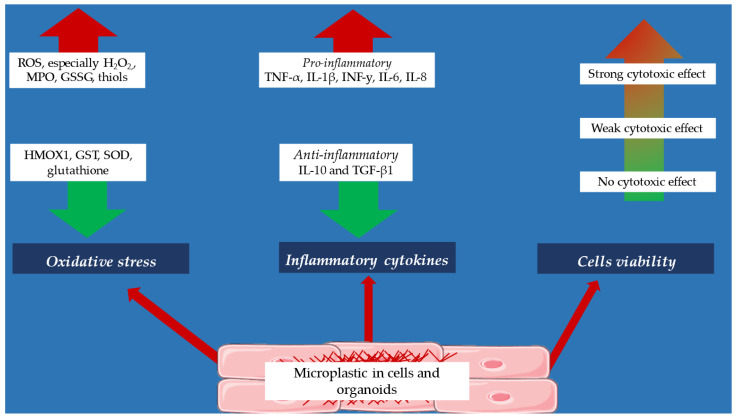
Biochemical and cellular effects of microplastics.

A summary of the biochemical cellular properties of microplastics is shown in Figure 4 and in Table 1.

Figure 4 provides a visual representation of the biochemical and cellular effects of microplastics, summarizing key pathways involved in inflammation, oxidative stress, and cytotoxicity. The following table presents a detailed breakdown of these effects across different cell types and biological systems.

Current findings indicate that microplastics contribute to immune dysregulation by increasing pro-inflammatory cytokine expression while simultaneously suppressing anti-inflammatory mediators [45,47,53]. This imbalance in immune signaling suggests a potential role of microplastic-induced inflammation in chronic disease progression [47,48,50]. Additionally, oxidative stress markers such as ROS and nitric oxide show increased expression under microplastic exposure [51,54,55,56,57], while key antioxidant defenses such as glutathione and glutathione reductase activity decline, exacerbating cellular vulnerability [58,59,60]. These findings highlight the need for further research to determine thresholds for toxicity, the mechanistic pathways involved, and how microplastic composition affects biological responses [59,61].

## 8. Biomonitoring of Microplastic Exposure

Biomonitoring is essential for understanding the extent of human exposure to microplastics and its potential health implications. Several studies have demonstrated the ability to detect MPs in human blood, urine, lung tissue, breast milk, and placental samples, confirming that microplastics can enter and persist within the human body. To improve detection accuracy, various analytical techniques have been employed:

- Flow cytometry, a laser-based method, has been increasingly used for identifying MPs in human fluids. By applying fluorescent staining agents such as Nile red, this approach enables rapid classification of MP particles based on their optical properties [64].
- Pyrolysis-GC/MS, which facilitates detailed compositional analysis of MPs in biological samples such as urine and plasma [65].
- Raman and Fourier-transform infrared (FTIR) spectroscopy, commonly utilized for determining polymer composition in human tissues [66].
- Microscopy-based techniques, including fluorescence and electron microscopy, have been applied to visualize MP accumulation in placenta and breast milk, raising concerns about maternal and fetal exposure [67].

Microplastics were identified in 77% of analyzed blood samples, indicating their potential systemic circulation within the human body [68]. Additionally, their presence was detected in 12 out of 16 human placentas, raising concerns about fetal exposure and possible transplacental transfer [67]. Inhalation has also been suggested as a significant exposure pathway, with 88% of lung biopsy specimens showing microplastic accumulation through Raman spectroscopy analysis [65]. Furthermore, dietary intake appears to be a persistent source of exposure, as microplastics were found in 100% of tested stool samples, confirming their presence in individuals across various regions [66,69].

As biomonitoring methodologies continue to advance, standardization of protocols and exposure thresholds will be critical for accurately assessing human health risks associated with MP contamination.

## 9. Policy and Regulation

To effectively address the issue of microplastic pollution from smoking products, comprehensive policies and regulations are necessary. The recent regulatory actions have focused on limiting the presence of microplastics in consumer goods, including cigarette filters. The European Union’s Single-Use Plastics Directive (SUPD) has introduced measures to reduce plastic waste from tobacco products, requiring manufacturers to contribute to cleanup costs and implement clearer labeling on cigarette packaging.

From a public health surveillance perspective, studies have highlighted the increasing detection of microplastics in lung tissue, raising concerns about their potential role in respiratory diseases. The WHO Global Tobacco Epidemic Report (2023) emphasizes the need for stricter monitoring of pollutants associated with smoking, including microplastics [70].

Furthermore, biomonitoring initiatives have been proposed to track microplastic exposure in smokers, with recent findings suggesting that inhaled microplastics may contribute to oxidative stress and inflammatory responses in lung tissue [68].

These regulatory and public health measures underscore the growing recognition of microplastic pollution as a significant environmental and health concern, necessitating continued research and policy development. In response to the above problem, governments and regulatory bodies can implement concrete measures to reduce the environmental impact of smoking-related plastic waste, including the following:

- Banning or restricting the use of non-biodegradable cigarette filters: Encouraging the development and adoption of biodegradable alternatives could significantly reduce the amount of plastic waste generated by cigarette butts.
- Implementing Extended Producer Responsibility (EPR) programs: Manufacturers of smoking products could be held accountable for the environmental impact of their products through EPR schemes. This could involve funding waste management programs, recycling initiatives, and public education campaigns.
- Strengthening waste management infrastructure: Improving the availability and accessibility of disposal options for smoking-related waste can help prevent littering and reduce plastic pollution.
- Raising public awareness: Public health campaigns can educate smokers about the environmental and health risks associated with microplastics in smoking products, encouraging responsible disposal practices and reducing tobacco use [70].

## 10. Discussion

The findings from the studies reviewed indicate a concerning relationship between microplastics, smoking, and inflammation. The degradation of traditional cigarettes contributes to the release of microplastics into the environment. These microplastics can enter the human body through inhalation, ingestion, or via skin, leading to various health issues, including inflammation and oxidative stress.

The studies demonstrate that microplastics can disrupt immune responses, particularly in the liver and gut. The polarization of macrophages and the infiltration of natural killer cells suggest that microplastics can alter the immune landscape, potentially leading to chronic inflammation. This is particularly concerning given the established link between chronic inflammation and various diseases, including cancer and cardiovascular diseases.

Moreover, the impact of microplastics on reactive oxygen species (ROS) production highlights the potential for oxidative stress to contribute to cellular damage. The studies indicate that exposure to microplastics can lead to an imbalance in antioxidant defenses, resulting in increased oxidative stress. This oxidative stress can further exacerbate inflammation and contribute to the development of various health conditions.

Future research should focus on elucidating the mechanisms by which microplastics impact human health and exploring effective strategies for mitigating their presence in the environment [71,72]. Collaboration between governments, industries, and communities is essential to protect public health and preserve the planet for future generations [65].

To mitigate the impact of microplastics from smoking products, several strategies can be employed. First, public awareness campaigns can educate smokers about the environmental consequences of improper disposal of cigarette butts and vaping devices. Encouraging responsible disposal practices, such as using designated waste bins, can help reduce the amount of plastic waste entering the environment [72,73,74].

Second, the development of biodegradable alternatives to traditional cigarette filters could significantly reduce the contribution of microplastics to pollution. Research into materials that can effectively filter smoke while being environmentally friendly is essential Additionally, implementing stricter regulations on the production and disposal of smoking products can help hold manufacturers accountable for their environmental impact [69,70]

What is more, promoting smoking cessation programs can reduce the overall consumption of tobacco products, thereby decreasing the volume of cigarette waste generated. Effective smoking cessation interventions, particularly those targeting youth and vulnerable populations, can contribute to reducing smoking prevalence and its associated environmental impacts [75,76]

Ultimately, it is essential to understand the interplay between inflammation, oxidative stress, and cytotoxicity, which creates a complex biological response to microplastic exposure. Although each mechanism has been studied independently, it is crucial to understand how these processes interact and influence each other. This interconnected relationship is further examined in the sections below.

### 10.1. Inflammatory Response: Conflicting Findings on Cytokine Modulation

Microplastic exposure has been shown to upregulate pro-inflammatory cytokines, such as TNF-α, IL-6, and IL-8, across different cell models, reinforcing its role in chronic inflammation [45,46,47]. However, an opposing trend emerges with anti-inflammatory cytokine suppression, particularly IL-10 and TGF-β, which may prevent resolution of inflammation and drive persistent tissue damage [48,50,53]. The contradictory nature of these responses suggests that microplastics may disrupt immune homeostasis, creating a prolonged inflammatory state without appropriate regulation. Understanding whether this imbalance predisposes individuals to inflammatory disorders remains a critical research avenue.

### 10.2. Oxidative Stress Paradox: Increased ROS Production vs. Adaptive Responses

Multiple studies confirm that microplastic exposure triggers oxidative stress, increasing ROS, nitric oxide, and H_2_O_2_ levels in various cell types [53,54,55,56]. However, certain biological systems display compensatory antioxidant mechanisms, such as increased thiol group expression and enzymatic activity, which may act as a defense against oxidative damage [50]. The existence of both heightened oxidative stress and protective cellular responses raises questions about whether short-term exposure induces cellular adaptation while long-term accumulation overwhelms detoxification pathways. Further studies should distinguish acute vs. chronic exposure effects to determine how oxidative stress contributes to disease progression and cellular aging [59,60].

### 10.3. Cytotoxicity Discrepancies: Material-Dependent Toxicity

The cytotoxic impact of microplastics remains highly variable across studies, largely dependent on plastic type, concentration, and exposure duration [49,54,56,59,60]. For example, polystyrene induces apoptosis and necrosis, while polyethylene microparticles exhibit no cytotoxicity in certain models [56,62]. Additionally, PVC microplastics appear to be highly toxic to lymphocytes, suggesting that some plastic formulations may pose greater health risks than others [63]. These discrepancies indicate the necessity of standardized cytotoxicity assays, ensuring that differences in methodology, plastic composition, and biological models do not confound results or oversimplify conclusions.

### 10.4. Study Quality Assessment: Standardization Challenges

While the discussion synthesizes existing findings, variability in methodologies remains a barrier to definitive conclusions on microplastic toxicity. Studies differ in cell types, exposure conditions, and endpoint measurements, complicating direct comparisons. Implementing standardized experimental protocols, including consistent plastic particle characterization, exposure duration, and cellular response metrics, will be essential in refining the scientific consensus on microplastic toxicity [60,62].

## 11. Future Perspectives

Alternative forms of smoking such as e-cigarettes and heated tobacco products are becoming increasingly popular. E-cigarettes are non-flammable tobacco products that mimic classic tobacco smoking, but without the burning of tobacco [73]. Globally, there has been an increase in e-cigarette users, especially among young people. Initially, e-cigarette smoking was advocated as a healthy alternative to classic cigarettes. However, their exact effects on human health are unknown. Another form of tobacco use is heated tobacco products (HTPs). These products allow nicotine to be inhaled by heating the tobacco (350 °C) instead of burning it at high temperatures [77,78]. Like e-cigarettes, their exact effects on human health are currently unknown [77,78].

Currently, there are no data that would determine whether e-cigarettes and HTPs are sources of microplastics. It is postulated that users of these forms of smoking introduce microplastic particles into the body. However, since the exact effects of both these forms of smoking and microplastic itself on human health are currently unknown, it is advisable to conduct future studies to determine whether these products are sources of microplastic, what the relationship between their use and potential human health risks is, and whether the body’s homeostasis could potentially be affected by the microplastic they contain.

Another issue that is closely related to microplastics is brain physiology. The potential impact of microplastics on neurological health is an emerging area of research. Microplastics can cross the blood–brain barrier, leading to potential neurotoxicity. Studies have demonstrated that exposure to microplastics can result in behavioral changes, cognitive impairments, and neuroinflammation in animal models [56,79]. The accumulation of microplastics in brain tissues raises concerns about their long-term effects on mental health and neurological diseases. Further research is needed to understand the mechanisms by which microplastics impact the nervous system and the potential risks for human health.

Smoking has long been recognized as a risk factor for mental health, leading to increased vulnerability to depression and anxiety disorders. Nicotine temporarily improves mood through its effects on the dopaminergic system, but long-term, it can lead to neurotransmitter dysregulation, exacerbating depressive symptoms. At the same time, studies have indicated severe structural and functional changes in the brain of smokers, including inflammation and oxidative stress, which increase the risk of neurodegeneration and neuropsychiatric disorders such as schizophrenia and dementia [80,81].

Also of concern is the impact of microplastics, whose particles can penetrate the brain, increasing inflammation and increasing the risk of neurodegeneration. It has been established that toxins present in cigarette smoke can weaken the blood–brain barrier, facilitating the penetration of microplastic into neural tissue.

In summary, the synergistic effects of cigarette smoking and microplastic exposure may contribute to severe cognitive impairment and increased risk of depression [81,82,83]. It is therefore important to conduct further research on this issue to determine the mechanisms of these harmful interactions and their long-term effects on public health.

Beyond the physical health impacts, the presence of microplastics in smoking products can have psychological and social implications. Awareness of microplastic contamination may influence individuals’ perceptions and behaviors related to smoking. Public health campaigns highlighting the environmental and health risks associated with microplastics in smoking products could contribute to changing attitudes and reducing tobacco use [66]. Additionally, the social stigma associated with contributing to plastic pollution might motivate individuals to seek alternatives or quit smoking altogether.

Assessing human exposure to microplastics (MPs) remains a developing area of research, with no universally standardized protocols currently available. However, emerging studies suggest that such exposure can be evaluated through direct and indirect detection methods.

Direct detection methods involve identifying MPs directly in biological samples such as plasma, serum, urine, saliva, or whole blood, using advanced analytical techniques. Pyrolysis–gas chromatography/mass spectrometry (Pyr-GC/MS) has been proposed as a viable approach for detecting and quantifying MPs in human fluids [65]. Indirect biomarkers of exposure rely on measuring physiological indicators such as cytokine levels, chemokines, and oxidative stress markers, which may signal an immune or inflammatory response to MP presence [67]

Although these methodologies offer promising directions, further research is required to validate their effectiveness, establish exposure thresholds, and standardize analytical procedures for consistent assessment across populations.

## 12. Conclusions

This comprehensive literature review highlights the hidden threats posed by microplastics in traditional cigarettes. The presence of microplastics in smoking products raises significant concerns for both human health and environmental sustainability. Microplastics can induce oxidative stress and inflammation, leading to various health issues, including respiratory and digestive diseases and potential cancer risks. The environmental impact of microplastics extends to aquatic and terrestrial ecosystems, contributing to plastic pollution and affecting biodiversity. Addressing the issue of microplastic pollution from smoking products requires a multifaceted approach, including public awareness campaigns, the development of biodegradable alternatives, stricter regulations, and improved waste management practices. Future research should focus on elucidating the mechanisms by which microplastics impact human health and exploring effective strategies for mitigating their presence in the environment. Collaboration between governments, industries, and communities is essential to protect public health and preserve the planet for future generations.

A deeper understanding of the interplay between inflammation, oxidative stress, and cytotoxicity is crucial for assessing the long-term consequences of microplastic exposure. Research suggests that these biological responses are not isolated but rather interdependent, influencing cellular homeostasis in complex ways.

For instance, chronic inflammation, characterized by heightened TNF-α and IL-6, can exacerbate oxidative stress, leading to increased ROS production and mitochondrial dysfunction [45,46,50,53]. Similarly, oxidative stress-induced DNA damage may contribute to cytotoxic effects, promoting apoptosis and necrosis in vulnerable cells [49,59,60]. This cyclical interaction underscores the necessity of a holistic approach that examines these effects collectively, rather than treating them as distinct phenomena.

Moreover, the variability in immune responses and oxidative defenses between different cell types suggests that individual susceptibility to microplastic-induced toxicity may depend on genetic and environmental factors. Standardized studies evaluating dose-dependent responses and exposure thresholds will be essential in determining the mechanistic pathways leading to adverse health outcomes [57,58,61].

Integrating these insights into future research strategies will be fundamental in developing intervention measures, improving biomonitoring techniques, and guiding regulatory policies aimed at mitigating microplastic contamination.

## Figures and Tables

**Table 1:** Effect of microplastics on inflammatory cytokines, oxidative stress parameters, and cells viability.

Effect of micropastics on inflammatory cytokines
↑ produce of IL-6, TNF-α, IL-8, IL-10 (monocytes, dendritic cells) [44,45]
↑ produce of IL-6, TNF-α, IL-8, IL-10 (epithelial cells) [47]
↑ produce of TNF-α, IL-1β, INF-y, IL-6, IL-8 (cancer cell lines—HeLa, A549, AGS, THP-1, HaCaT, U937 [49,50]
↑ produce of TNF-α, IL-1β, INF-y, IL-6, IL-8 (kidney of a human embryo) [50]
↑ TNF-α, IL-6, IL-1β expression (microglia) [46]
↓ TGF-β and IL-10 expression (microglia) [46]
↑ IFN-γ, TNF-α, IL-1β, IL-6, and IL-33 expression (liver tissue) [53]
↓ IL-4, IL-5, IL-10, IL-18, and TGF-β1 expression (liver tissue) [53]
Effect of micropastics on oxidative stress parameters
↑ produce of ROS (cancer cell lines—U937 cells, THP-1, A549, HaCaT, Caco-2 and HeLa cells [50,53,54,55]
↑↑ produce of H_2_0_2_ (Caco-2 cells) [54]
↑ produce of nitric oxide (monocytes) [45]
↑ amount of thiol groups (kidney of a human embryo) [48]
↓ amount of glutathione (lymphocytes, liver and heart organoids) [57,59,60]
↑ production of myeloperoxidase (monocytes, dendritic cells) [45]
↑ glutathione disulfide activity (lymphocytes) [58]
↓ heme oxygenase activity (HEK293 cells) [58]
↓ glutathione reductase activity (liver organoids) [60]
↑ superoxide dismutase activity (liver and hearts organoids) [59,60]
Effect of micropastics on cells viability
-induction of apoptosis and necrosis (cancer cell lines, human-originated cardiac organoids) [48,59,60]
-weak cytotoxicity effect (cancer cell lines—A549, HEK293, HeLa, AGS) [54,56]
-weak cytotoxic effect in concentration (kidney of a human embryo) [50]
-no cytotoxic effect (U937 cells, THP-1, A549, HaCaT, Caco-2, T98G and HeLa cells) [55,61]
-cytotoxic effect (lymphocytes) [64]

↓: decrease; ↑ increase.

## Abbreviations

The following abbreviations are used in this manuscript:

AGS cells	Human gastric adenocarcinoma cell line;
AGS	Human gastric adenocarcinoma cell line;
Caco-2	Human colorectal adenocarcinoma cell line;
GSSG	Glutathione disulfide;
GST	Glutathione reductase;
H_2_O_2_	Hydrogen peroxide;
HaCaT	Spontaneously immortalized human keratinocyte cell line;
HEK293	Human embryonic kidney cell line;
HeLa	Epithelial cervical cancer cell line;
HMOX1	Heme oxygenase 1;
IL-10	Interleukin 10;
IL-1β	Interleukin beta 1;
IL-6	Interleukin 6;
IL-8	Interleukin 8;
INF-y	Interferon gamma;
MP	Microplastic;
MPO	Myeloperoxidase;
NF-κB	Nuclear Factor-Kappa B;
PE	Polyethylene;
PMMA	Polymethylmethacrylate;
RedOx	Reduction–oxidation;
ROS	Reactive oxygen species;
SOD	Superoxide dismutase;
T98G	Glioblastoma cell line;
TGF-β1	Transforming growth factor beta 1;
THP-1	Human leukemia monocytic cell line;
TNF-α	Tumor necrosis factor α;
U937	Human pro-monocytic leukemia cell line
